# Traditional Chinese medicine injections with activating blood circulation, equivalent effect of anticoagulation or antiplatelet, for acute myocardial infarction: A protocol for the systematic review and meta-analysis of randomized clinical trials

**DOI:** 10.1097/MD.0000000000029089

**Published:** 2022-06-17

**Authors:** Xiang Li, Yan Lou, Ju-Ju Shang, Hong-Xu Liu, Jia-Ping Chen, Hui-Wen Zhou

**Affiliations:** Department of Cardiology, Beijing Hospital of Traditional Chinese Medicine affiliated to Capital Medical University, Beijing, China.

**Keywords:** activating blood circulation, acute myocardial infarction, protocol, systematic review, Traditional Chinese medicine injections

## Abstract

**Background:**

In spite of a growing number in the use of percutaneous coronary intervention (PCI) in China, the mortality of acute myocardial infarction (AMI) has not decreased. Traditional Chinese medicine injections for Activating Blood Circulation (TCMi-ABC), equivalent effect of anticoagulation or antiplatelet, are widely used in China; however, the improvement of fatality towards AMI is unclear. Therefore, we intend to conduct a systematic review and meta-analysis to evaluate the efficacy and safety of TCMi-ABC in treatment with AMI.

**Methods:**

Based on the “National Medical Products Administration of China,” TCMi-ABC with AMI treatment indication will be selected, including Danhong injection, Sodium Tanshinone IIA Sulfonate injection, Danshen Chuanxiongqin injection, and Puerarin injection. Randomized controlled studies will be searched from as follows: PubMed, Embase, the CENTRAL in Cochrane Library, Chinese Biomedical Literature Database (SinoMed), China National Knowledge Infrastructure (CNKI), China Science and Technology Journal Database (VIP), and Wanfang Data Knowledge Service Platform. Two researchers will work independently on literature selection, data extraction, and quality assessment. The outcomes focus on the effects of TCMi-ABC on fatality of patients with AMI in hospitalization and in the long term, the incidence of malignant arrhythmia, left ventricular ejection fraction, and adverse events. RevMan 5.4.1 software was used for mate analysis.

**Results:**

This study will conduct a comprehensive literature search and provide a systematic synthesis of current published data to explore the efficacy and safety of TCMi-ABC for AMI.

**Conclusion:**

This study will provide high-quality evidence for treatment of AMI with TCMi-ABC in terms of efficacy and safety, which may help clinicians make a better complementary treatment schedule of patients with AMI.

## 1 Introduction

### 1.1 Description of the condition

*Global Burden of Disease Study 2019* showed that acute myocardial infarction (AMI) was one of the major diseases with high global morbidity and mortality, causing a huge global economic and medical burden,^[1]^ which was also reflected in China. According to the “*Report on Cardiovascular Health and Diseases Burden in China: an Updated Summary of 2020*,” the overall mortality of AMI in China from 2002 to 2018 showed an upward trend, especially a rapid increase in 2005.^[2]^ Recently, percutaneous coronary intervention (PCI) has been rapidly developed in China, as is reported that the number of cases of PCI in China received more than 915,000 in 2018, which had surpassed the United States and ranked first in the world. But *China PEACE Studies*^[3]^ showed that in China the hospital fatality of AMI has not decreased significantly as the growing development of PCI. In addition to popularizing secondary prevention of coronary heart disease actively and promoting early reperfusion treatment of AMI, whether traditional Chinese medicine (TCM) as a complementary method can be beneficial in reducing the fatality of patients with AMI has received more and more attentions, especially since artemisinin was recognized as a successful treatment for malaria as a Nobel Prize.

TCM has a history of treating AMI for more than 2000 years. Translating the “experience” of TCM in treatment with AMI into “evidence” is regarded as a key step to help more patients with AMI to get clinical benefits. In the 1970s, Professor Ke-Ji Chen discovered a type of Chinese medicine with function of anticoagulant and antiplatelet on the basis of reading a large number of ancient Chinese medicine books, which calls “Activating Blood Circulation (ABC)” drugs, can play a role in myocardial protection. In the 1980s, Professor Chen conducted a first evaluation of Chinese medicine formulae with function of ABC called Guanxin No. 2 in treatment with stable angina by a multicenter, randomized, double-blind, placebo-controlled clinical trial, which showed that Guanxin No. 2 had myocardial protective effects including relieving angina, improving myocardial ischemia and abnormal hemorheology.^[4]^ As the research went on, it has gradually been discovered that the “ABC” drugs with equivalent effects of anticoagulant and antiplatelet can be beneficial in reducing the incidence of cardiovascular events and the risk of restenosis in coronary stents.^[5,6]^ At present, “ABC” drugs have become the most commonly used Chinese herbal medicine for coronary heart disease in Chinese mainland.

### 1.2 Description of the intervention

Strategies of western medicine in treatment with AMI include general treatments, reperfusion therapy, and drug therapy. General treatments refer to measures such as monitoring of vital signs, relief of pain, breathing difficulties and anxiety, and improvement of hypoxia. Drug treatment mainly includes antiplatelet aggregation drugs, anticoagulant drugs, lipid-lowering drugs, β-receptor blockers, calcium channel blockers, nitrate drugs, angiotensin converting enzyme inhibitors or angiotensin II receptor. Reperfusion therapy is achieved through PCI and thrombolysis and coronary artery bypass surgery.^[7]^ Besides the current standardized Western medicine treatment, Traditional Chinese medicine injection (TCMI), as a complementary and alternative treatment, may play a positive role in further reducing the fatality of AMI.^[3]^

TCMI is a sterile preparation for injection after extraction and purification from traditional Chinese medicine.^[8]^ It is a new dosage form combined with TCM theory and modern pharmaceutical technology, which is widely used in China.^[9]^ The world's first TCMI, Bupleurum injection, came out in 1941 and has a history of 80 years. Currently, there have been 134 kinds of TCMIs on the market in China,^[10]^ in which also play an important role in the treatment of AMI.^[11]^ A survey involving more than 5000 AMI patients in 26 Level three Class A TCM hospitals in China during 10 years period from 1999 to 2008 showed that TCMIs had the potential to reduce the fatality of AMI patients,^[12,13]^ but lack of high-level evidence.

To seek high-level evidence for the efficacy and safety of Traditional Chinese medicine injections for Activating Blood Circulation (TCMi-ABC) in treatment with AMI, firstly we searched information about TCMi-ABC with indications for the treatment of AMI on the data platform of “National Medical Products Administration of China” (https://www.nmpa.gov.cn/). The search results included *Danhong injection*, *Tanshinone IIA sodium sulfonate injection*, *Danshen ligustrazine injection*, and *puerarin injection*. We will evaluate the clinical value of these TCMI-ABC interventions for AMI on the basis of the current standardized Western medicine treatment.

### 1.3 How the intervention might work?

*Danhong injection* (DHI) is an injection made from two Chinese herbal medicines: carthami flos and salvia miltiorrhiza bunge. *Tanshinone IIA sodium sulfonate injection (*TIIaI*)* is an effective compound extracted from an herb medicine called *Salvia Miltiorrhiza*. *Danshen ligustrazine injection* (DLI) is a compound injection composed of *Salviae miltiorrhizae* extract and ligustrazine hydrochloride monomer. *Puerarin injection* (PI) is an isoflavone compound extracted from an herb medicine called *Pueraria lobata*. Experimental studies have demonstrated these four TCMi-ABCs with multiple cardiac-protective effects on AMI, including prevention of ischemia reperfusion injury, improvement of myocardial metabolism, reduction of myocardial infarction area and functions of anti-oxidant stress and anti-inflammatory damage.^[14–27]^

### 1.4 Why it is important to do this review?

TCMi-ABC is widely used in China in treatment of coronary artery disease (CAD). As shown in previous researches, these four TCMi-ABCs with indications for the treatment of AMI can be used as a complementary method to play a role on myocardial protection. However whether they can safely and effectively reduce the fatality of AMI patients is still controversial. It is worth noting that a recent retrospective investigation conducted in mainland China suggested that TCMI had not been found benefits in the application of acute heart failure patients with CAD. On the contrary, *salvia miltiorrhiza bunge*, one of the most commonly used drugs for ABC in the treatment of AMI, had been found to increase the risk of bleeding in patients.^[28]^ Therefore, it is necessary to carry out a systematic review of the efficacy and safety of TCMi-ABC in treatment with AMI based on the current original research.

### 1.5 Objectives

To systematically evaluate the efficacy and safety of TCMi-ABC for AMI.

## 2 Methods

### 2.1 Study registration

The protocol was registered on INPLASY (https://inplasy.com/inplasy-2021-7-0082/) and its registration number was INPLASY202170082. We will complete this protocol according to the preferred reporting items for systematic reviews and meta-analysis protocols. The changes are described in our full review if needed.

### 2.2 Ethic approval

Ethical approval will not be necessary since this systematic review and meta-analysis only uses published papers, which will not reveal personal privacy and violate human rights.

### 2.3 Inclusion criteria for study selection

#### 2.3.1 Types of studies

Only randomized controlled trials can be included in our research, case reports, animal experiments, and reviews will be excluded, and there will be no language restrictions in the selection of literature.

#### 2.3.2 Types of patients

Regardless of their nationality, age, race, patients who meet the diagnostic criteria for AMI will be included as participants.

#### 2.3.3 Types of interventions

The experimental group was treated with one of these four TCMi-ABCs (including DHI, TIIaI, DLI, and PI) on the basis of western medicine treatment. The control group was treated with Western medicine treatment alone or combined with placebo. Western medicine basis treatments include: general treatment (including vital signs monitoring, symptom relief, etc), reperfusion therapy (including PCI, thrombolytic therapy, and coronary artery bypass grafting), drug therapy (including: antiplatelet, anticoagulant, lipid-lowering, etc). TCMi-ABC was the only intervention difference between the control group and the experimental group in all studies.

#### 2.3.4 Types of outcome measures

The outcomes focus on the effects of TCMi-ABC on fatality of patients with AMI in hospitalization and in the long term. The long-term fatality refers to the fatality of AMI 1 year or more after the disease. The other outcomes include the incidence of malignant arrhythmia, left ventricular ejection fraction (LVEF), adverse events (such as nausea, dizziness, and vomiting).^[29]^ The included studies reported at least one of the above outcomes.

### 2.4 Exclusion criteria

(1) Diagnostic criteria were not mentioned or unclear in the literature.
(2) The clinical trial control setting is unreasonable or does not meet the requirements of this study, such as the combination of other Chinese patent medicine in the control group.
(3) Duplicate publications.
(4) Literatures for the required effect indicators are not included.

### 2.5 Search methods for identification of studies

#### 2.5.1 Electronic searches

The search terms and strategy described in Additional File 1 will be used to search the following databases: PubMed, Embase, the CENTRAL in Cochrane Library, Chinese Biomedical Literature Database (SinoMed), China National Knowledge Infrastructure (CNKI), China Science and Technology Journal Database (VIP),Wanfang Data Knowledge Service Platform. All database retrieval times are selected from the date of database establishment. Taking PubMed as an example, the initial search strategy is shown in Table 1, which will be adjusted according to the specific database.

**Table 1:** Search strategy of the PubMed.

Search	Query
#1	Myocardial infarction[MeSH]
#2	Myocardial infarction OR acute myocardial infarction OR AMI OR infarction, myocardial OR cardiovascular stroke OR infarctions, myocardial OR myocardial infarctions OR cardiovascular strokes OR heart attack OR strokes, cardiovascular OR myocardial infarct OR infarct, myocardial OR stroke, cardiovascular[Title/Abstract]
#3	#1 OR #2
#4	Injection[Mesh]
#5	Injection OR injectables OR injectable OR injections[Title/Abstract]
#6	#4 OR #5
#7	Puerarin OR Danhong OR Dan red OR Sulfotanshinone sodium OR Sodium Tanshinone IIA Sulfonate OR Tanshinone IIa OR *Salviae miltiorrhizae* and ligustrazine hydrochloride injection OR Danshen Chuanxiongqin[Title/Abstract]
#8	Randomized controlled trial[publication type] OR Controlled Clinical Trial[Publication Type] OR random∗[All Fields]
#9	#3 AND #6 AND #7 AND #8

AMI = acute myocardial infarction.

#### 2.5.2 Searching other resources

In addition to the literatures retrieved from the electronic databases, our study will also search for literatures whose titles may be relevant about it in the bibliography attached to the included literatures. Paper books and journals related to our study will be manually reviewed to ensure that the included literature is comprehensive. Related ClinicalTrials will also be searched at “ClinicalTrials.gov.” to determine whether any literatures to be published met the inclusion criteria.

### 2.6 Search methods for identification of studies

#### 2.6.1 Selection of studies

**Figure 1:**
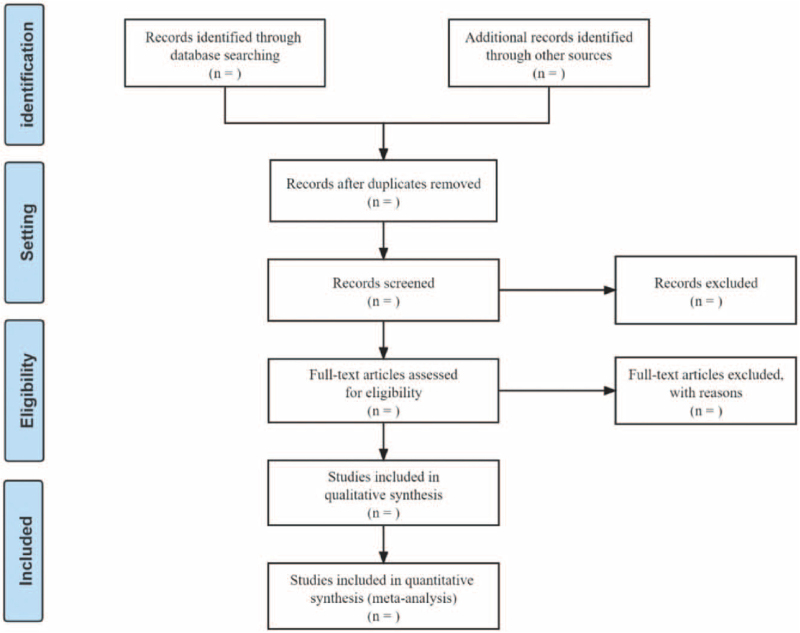
PRISMA flow diagram of the study selection process.

Each step of the literature search will be performed independently by 2 reviewers according to the established search rules. After screening based on the inclusion criteria, 2 reviewers (JPC and HWZ) will exclude the papers that do not meet the inclusion criteria by reading the titles and abstracts. Then, the reviewers will check the full texts to determine the final decision according to the criteria. If the articles information is insufficient, we will try to contact the authors to obtain the necessary details. When 2 reviewers have different opinions, the final decision will be made by the third reviewer (YL). The selection flow process of is shown in the preferred reporting items for systematic reviews and meta-analysis flow chart (Fig. 1).

#### 2.6.2 Data extraction and management

First we design an extraction form that meets the purpose of this system review, which will include the following information from the included studies: participant characteristics, interventions, outcomes, and adverse events. Two investigators (JPC and HWZ) independently completed the data collection form for all eligible studies. The corresponding authors will be contacted to request insufficient or missing information. Disagreements will be resolve by discussion or by appealing to a third author (YL). The data were stored in Microsoft Excel.

#### 2.6.3 Assessment of risk of bias in included studies

We will use the Cochrane risk assessment tool to assess the risk of bias. The methodological quality of RCTs will be independently evaluated by 2 reviewers (JPC and HWZ). The following 7 items will be included: random sequence generation, allocation concealment, blinding of participants and caregivers, blinding of outcome evaluator, incomplete outcome data, selective reporting, and other bias. High, low, and unclear assessments will be performed for each item. Any disagreement between the 2 reviewers (JPC and HWZ) will be resolved by a discussion. Further disagreements will be arbitrated by the third author (YL).

#### 2.6.4 Measures of treatment effect

We will use the mean difference or standard mean difference with 95% confidence intervals as the effect measure for continuous data. Dichotomous outcomes will be analyzed by the risk ratio with 95% confidence interval.^[30]^

#### 2.6.5 Dealing with missing data

When there are events in the reports that are unclear or do not report data, we will contact the author by phone or email to obtain complete information.^[31]^

#### 2.6.6 Assessment of heterogeneity and data synthesis

##### 2.6.6.1 Assessment of heterogeneity

We will use RevMan 5.4.1 software to detect the heterogeneity between studies. When *P* < .01, I^2^ > 50%, there is significant heterogeneity between studies; otherwise, heterogeneity is acceptable.

##### 2.6.6.2 Data synthesis

RevMan 5.4.1 will be used for all statistical analyses. We used the random effects model to merge the data. The results of the meta-analyses are presented as forest plots. When the results are unsuitable for combination due to the clinical or methodological heterogeneity, we will perform a descriptive analysis.

#### 2.6.7 Sensitivity analysis

If the result shows high heterogeneity (the I^2^ test is >50%), we will conduct a sensitivity analysis. We will then acquire a stable result of our study.

#### 2.6.8 Subgroup analysis

If there are adequate studies and available data, we will conduct subgroup analysis for different syndrome types of insomnia to explain the heterogeneity among studies.

#### 2.6.9 Assessment of reporting biases

Funnel plots were used to explore the publication bias when 10 or more trials were included.

#### 2.6.10 Grading the quality of evidence

The certainty of a body of evidence will be assessed by using the approach developed by the Grades of Recommendation, Assessment, Development and Evaluation Working Group, involving risk of bias, heterogeneity, indirectness, imprecision, publication bias, and other domains. The certainty level will be rated as high, moderate, low, or very low, and the strength of evidence recommendation will be judged as strong or weak.

## 3 Discussion

Our systematic review will review the efficacy and safety of TCMi-ABC in the treatment of AMI comprehensively. The evidence from this review may benefit patients with AMI and clinicians, which will also help the formulation of relevant clinical guidelines. However, if there is a large degree of heterogeneity in method quality and outcome measurement, it may cause some challenges to the research results.

## Author contributions

XL and YL drafted the protocol. XL and JJS designed the search strategy. YJJ and HXL provided statistical expertise. JJS read, provided feedback, and approved the final manuscript. JPC, HWZ, and YL reviewed and revised the manuscript. All authors have read and approved the final version of the manuscript.

**Data curation:** Jia-Ping Chen, Hui-Wen Zhou.

**Funding acquisition:** Ju-ju Shang.

**Methodology:** Hong-Xu Liu.

**Writing – original draft:** Xiang Li, Yan Lou.
